# American dog ticks along their expanding range edge in Ontario, Canada

**DOI:** 10.1038/s41598-022-15009-9

**Published:** 2022-06-30

**Authors:** Mark P. Nelder, Curtis B. Russell, Steven Johnson, Ye Li, Kirby Cronin, Tania Cawston, Samir N. Patel

**Affiliations:** 1 Enteric, Zoonotic and Vector-Borne Diseases; Health Protection, Operations and Response, Public Health Ontario, Toronto, ON Canada; 2 Informatics, Knowledge Services, Public Health Ontario, Toronto, ON Canada; 3 Dalla Lana School of Public Health, University of Toronto, Toronto, ON Canada; 4 Public Health Ontario Laboratory, Public Health Ontario, Toronto, ON Canada; 5 National Microbiology Laboratory, Public Health Agency of Canada, Winnipeg, MB Canada; 6 Department of Laboratory Medicine and Pathobiology, University of Toronto, Toronto, ON Canada

**Keywords:** Ecology, Diseases

## Abstract

The American dog tick, *Dermacentor* *variabilis*, is a tick of public and veterinary health importance in North America. Using passive tick surveillance data, we document distribution changes for the American dog tick in Ontario, Canada, from 2010 through 2018. *Dermacentor* *variabilis* submissions from the public were geocoded and aggregated—from large to small administrative geographies—by health region, public health unit (PHU) and Forward Sortation Area (FSA). PHU hot spots with high rates of *D*. *variabilis* submissions were (1) Brant County, Haldimand-Norfolk and Niagara Regional in the Central West region and (2) Lambton and Winsor-Essex County in the South West region. The number of established *D*. *variabilis* populations with ≥ 6 submissions per year increased significantly during the study at regional (PHUs: 22 to 31) and local (FSAs: 27 to 91) scales. The range of *D*. *variabilis* increased similarly to the positive control (*Ixodes* *scapularis*) during the study and in contrast to the static range of the negative control (*Ixodes* *cookei*). Submission hot spots were in warmer, low elevation areas with poorly drained soils, compared to the province’s low submission areas. *Dermacentor* *variabilis* is spreading in Ontario and continued research into their vector ecology is required to assess medicoveterinary health risks.

## Introduction

The American dog tick, *Dermacentor* *variabilis*, is a temperate and sub-tropical species found throughout central and eastern USA, extending south into Mexico, with an isolated population along the American west coast^1^. In Canada, the American dog tick occurs in the southern portions of Saskatchewan, Manitoba, Ontario, Quebec and Nova Scotia^2^. Typically, *D*. *variabilis* occurs in deciduous forest ecotones such as fields, forest edges, trails, roadsides and brushy areas along waterways in rural, suburban and urban landscapes^1,3^. The American dog tick is a three-host tick, with larvae and nymphs feeding on rodents, and adults feeding on medium- to large-sized mammals. Reported hosts of *D*. *variabilis* in Ontario are cats (*Felis* *catus*), coyotes (*Canis* *latrans*), dogs (*Canis* *lupus* *familiaris*), horses (*Equus* *caballus*), humans (*Homo* *sapiens*), meadow voles (*Microtus* *pennsylvanicus*), North American porcupines (*Erethizon* *dorsatum*), northern raccoons (*Procyon* *lotor*), southern red-backed voles (*Myodes* *gapperi*), striped skunks (*Mephitis* *mephitis*), Virginia opossums (*Didelphis* *virginiana*) and white-footed mice (*Peromyscus* *leucopus*)^4–9^. Given the liberal host range of adult *D*. *variabilis*, distribution is limited mostly by the ecological requirements (e.g., appropriate habitat, humidity and temperature) of off-host ticks during diapause, egg development, host seeking, molting and oviposition^10^.

*Dermacentor* *variabilis* transmits several pathogens of public and/or veterinary health concern, including *Cytauxzoon* *felis* (cytauxzoonosis), *Francisella* *tularensis* (tularemia) and *Rickettsia* *rickettsii* (Rocky Mountain spotted fever)^2,11^. *Dermacentor* *variabilis* is a competent vector of *Anaplasma* *marginale* (bovine anaplasmosis), but transmission by American dog ticks was not demonstrated during a recent outbreak in Manitoba^12^. Furthermore, neurotoxins in the American dog tick’s saliva can cause tick paralysis in dogs, horses and humans^2,13^. *Dermacentor* *variabilis* is primarily a biting pest in Ontario, but we know little concerning the vector ecology of the American dog tick in the province. 

Despite an apparent low risk of *D*. *variabilis*-borne disease in Ontario, climate and land use changes can alter habitat-host-vector-pathogen dynamics^14,15^. Increases in suitable climate and habitat can amplify disease risks, by increasing tick population numbers and their distribution and lengthening the active season of ticks and their hosts^16^. To our knowledge, *D*. *variabilis* occurrence in Ontario was first reported in 1910, and until at least the 1980s, collection records were sporadic and concentrated south of Toronto (≈ 43.5° N)^17–20^. Recent work revealed the American dog tick’s range is expanding in Manitoba and Saskatchewan, potentially increasing the risk of disease from *D*. *variabilis*-borne pathogens^21^. The American dog tick is the second most commonly encountered tick in Ontario, behind the blacklegged tick (*Ixodes* *scapularis*), yet changes in *D*. *variabilis* distribution in the province remain unexplored^6^. The gold standard for determining a tick’s distribution is active surveillance through host trapping and tick dragging; however, this approach is not feasible given Ontario’s size of approximately 1 million km^2^. Accordingly, we describe *D*. *variabilis* distribution in Ontario using passive tick submissions from the public and determine if there has been a change in American dog tick distribution from 2010 through 2018.

## Materials and methods

### Study location

Ontario has a population of approximately 14 million and is located in the Great Lakes region of North America. Most of southern Ontario experiences a moderate continental climate and lies within the Mixedwood Plains Ecozone (≈ 83,000 km^2^); field and agricultural land comprise 59% of land cover in this ecozone (see citation for details of Ontario’s ecozones)^22,23^. Oaks, maples, yellow birch, ashes, eastern hemlock, American beech, American elm, basswood, wild black cherry, hickories, eastern white pine, firs and spruce dominate forested areas of southern Ontario^22,23^. In successional habitats, common plants include staghorn sumac, highbush cranberry, clover, red osier dogwood, goldenrod and willow^22,23^.

Currently, 34 public health units (PHUs) administer public health services in Ontario; however, for this study, we performed analyses with the previous 35-PHU classification that were in existence during the time frame of the study (for PHU acronyms and full names, see Supplementary Table S1). PHUs are further organized into seven health regions: Central East (DUR, HKP, PEL, PTC, SMD, YRK), Central West (BRN, HAL, HAM, HDN, NIA, WAT, WDG), Eastern (EOH, HPE, KFL, LGL, OTT, REN), North East (ALG, NPS, PQP, SUD, TSK), North West (NWR, THB), South West (CHK, GBO, HUR, LAM, MSL, OXE, PDH, WEC) and Toronto (TOR).

### Passive tick surveillance

We have described Ontario’s passive tick surveillance previously^24^. Briefly, Public Health Ontario (PHO) morphologically identifies ticks submitted by the public through healthcare providers or PHU offices. This study spanned from 2010 to 2018 and only included ticks submitted from human hosts. Tick submission data include the submitter’s postal code and travel history, along with tick submission date, stage, sex and number of ticks. The Forward Sortation Area (FSA) is the smallest geographic unit used in our analyses and is the first three characters of the six-character postal code of the submitter’s residence. There are 513 FSAs in Ontario with a median area of 24.7 km^2^ (interquartile range [IQR]: 8.7–151 km^2^; range: 0.3–408,433 km^2^), with geographically smaller FSAs in urban centers and larger FSAs in rural areas (see citation for further details on Ontario’s FSAs)^25^. The median area of the 35 PHUs was 3,806 km^2^ (interquartile range [IQR]: 2,036–8,988 km^2^; range: 630–266,291 km^2^). We used the submitter’s FSA to aggregate data to the PHU level and then to the larger health region level. Ticks potentially acquired outside of Ontario were excluded from analyses (*n* = 389). EOH, KFL and LGL stopped accepting tick submissions at their PHU offices in 2014 and HDN stopped in 2018; however, healthcare providers could still submit ticks from patients in these PHUs (for provincial summary, we included a subset of PHUs that excludes PHUs that ceased submissions during the study period).

We used existing criteria to determine if *D*. *variabilis* was established: a PHU or FSA had an established population if the public submitted at least six ticks in a year from that PHU or FSA. This criterion is based on research focusing on *I*. *scapularis* and *Ixodes* *pacificus* in the United States of America (USA) and applied at the county-level geography; the criterion has also been applied to *D*. *variabilis* and *Amblyomma* *americanum* in the USA (lone star tick)^26–29^. We compared *D*. *variabilis* with a relatively newly-established tick (*I*. *scapularis* = positive control; a species demonstrated as having an expanding range) and a long-established, nidicolous tick (*Ixodes* *cookei* = negative control; a species with a relatively static range)^6,30,31^. We also use the term occurrence to describe a tick’s distribution; occurrence is when the public submitted at least one tick from a PHU or FSA in a single year.

### Mapping and statistical analyses

We calculated provincial, health region and PHU submission rates of *D*. *variabilis* per 100,000 population (denominator = 2018 population data) using population data and estimates from Statistics Canada via IntelliHEALTH Ontario (October 19, 2017). We created distribution maps using Esri ArcGIS v10.3 (Esri; Redlands, California, USA; 2014) for tick submission rates for health regions and PHUs. We did not map submissions by FSA, as the high variability in FSA size makes mapping at this level of geography problematic (e.g., most urban FSAs are too small to see on a map and large, rural FSAs would dominate the map). In addition, rate of submission at the FSA-level was out-of-scope for this work since population data for FSAs (including yearly and monthly projections) were not available.

We modelled *D*. *variabilis* submission counts using a Poisson regression and the number of submissions as the dependent variable, including year (linear), health region or PHU and their interaction term with year to determine if there are different trends over time by geography (reported as relative risk [RR], 95% confidence interval [CI]). The model also accounted for seasonality with sine and cosine with periods of pi/3 and pi/6 on the 12 months within a year. Population for each month, year and PHU/health region are included as an offset term in the model on the log scale. To examine the geospatial spread of the ticks over time, we modelled the number of FSAs with at least one submission as outcome using Poisson regression, accounting for seasonality (same periods as the model above) and time (in year) without an offset term. R v3.6.2 (R Foundation for Statistical Computing; Vienna, Austria; 2019) was used for Poisson regression analyses (Supplemental Model Outputs). We used Excel v15.0 (Microsoft; Redmond, Washington, USA; 2013) and the Excel add-in Real Statistics Resource Pack v6.8 (Charles Zaiontz; www.real-statistics.com; 2013–2020) for all other analyses. To determine if there were changes in tick submission counts over time for PHUs and FSAs, we used simple linear regressions. We used a two-tailed Mann–Kendall test for changes in tick relative abundance, examining if the proportion of all tick submissions that were *D*. *variabilis*, *I*. *scapularis* or *I*. *cookei* changed over time at the provincial level. We made plots using Excel, except for Supplementary Figures S1, S2 and S5. For all analyses, we used a significance level of α = 0.05.

## Results

### Provincial summary

There were 17,434 (annual median = 1,901; IQR: 1,020–2,509) *D*. *variabilis* submissions made in Ontario (2010–2018), with a provincial submission rate of 124 per 100,000 population (Supplementary Table S1, S2). For a subset of PHUs (subset excludes PHUs that ceased submissions during the study period), 15,298 (annual median = 1704; IQR; 874–2,333) American dog tick submissions were made, with a submission rate of 109/100,000. The mean number of American dog ticks per submission was similar for all PHUs (mean ± SE = 1.08 ± 0.0039; *n* = 18,809) and the subset (1.08 ± 0.0042; *n* = 16,528) (t-test: *t* = 0.2, *P* = 0.79). For the remainder of the analyses, we will only report “all PHU” dataset results. For *I*. *scapularis*, 22,912 (annual median = 2,298; IQR: 1,966–3,245) submissions were made, with a submission rate of 163/100,000. For *I*. *cookei*, 2,324 (annual median = 241, IQR: 199–322) submissions were made, with a submission rate of 16.6/100,000.

**Figure 1:**
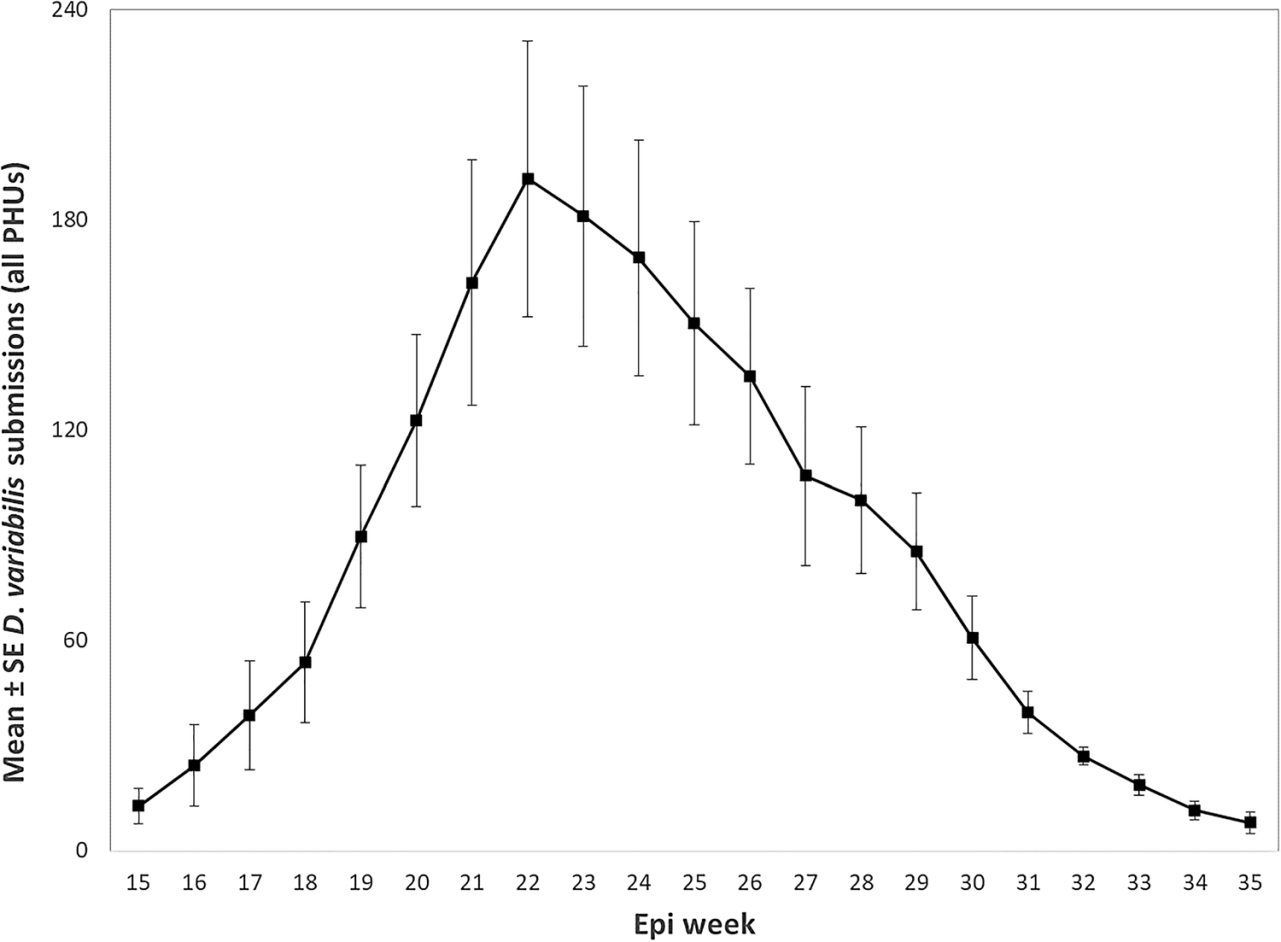
*Dermacentor* *variabilis* submissions per epidemiological week (epi week) for all public health units (PHUs) during the spring and summer: Ontario, Canada (2010–2018). Epi week 22 starts May 25–31, depending on year.

For *D*. *variabilis* submissions, 99.9% (17,414/17,428) were adult ticks, < 0.1% (*n* = 9) were nymphs and < 0.1% (*n* = 5) were mixed-stage submissions. For adult American dog tick submissions, 61.5% (10,717/17,414) were female, 36.4% (*n* = 6,334) were male and 1.9% (*n* = 337) were mixed-sex (i.e., sample contained male and female specimens). Submissions peaked during epidemiological week (epi week) 22 or approximately late May (Fig. 1).

Annual submission counts for *D*. *variabilis* (Supplementary Table S2) were correlated with submission counts of all three tick species combined (Pearson correlation: *r* = 0.96, *P* = 0.000038), *I*. *scapularis* (*r* = 0.95, *P* = 0.00010) and *I.* *cookei* (*r* = 0.94, *P* = 0.00016). The relative abundance of *D*. *variabilis* did not change significantly during the study (Mann–Kendall test: *Z* = 1.1, *P* = 0.25), similarly there was no change in relative abundance for *I*. *scapularis* (*Z* = -1.0, *P* = 0.35) and *I*. *cookei* (*Z* = -1.8, *P* = 0.076) (Table 1).

**Table 1:** Mann-Kendal test for changes in proportions (i.e., relative abundance) of all tick submissions that were *Dermacentor* *variabilis*, *Ixodes* *scapularis* (positive control), *Ixodes* *cookei* (negative control) and three species combined: Ontario, Canada (2010–2018).

Year	Percent of all tick submissions (%)			
	*Dermacentor* *variabilis*	*Ixodes* *scapularis*	*Ixodes* *cookei*	Three species combined
2010	37.9	48.8	7.2	93.9
2011	25.5	64.4	6.4	96.3
2012	34.2	56.7	4.5	95.4
2013	34.7	50.2	4.8	89.7
2014	27.6	51.7	5.9	85.2
2015	47.6	36.8	5.3	89.7
2016	41.1	44.1	4.3	89.5
2017	42.3	47.1	4.1	93.5
2018	37.6	50.8	5.2	93.6
Sen’s slope (95% CI)	1.1 (− 2.6, 3.1)	− 1.3 (− 4.2, 1.5)	− 1.8 (− 4.2, 0.1)	− 0.25 (− 1.7, 1.3)
*Z*-stat	1.1	− 1.0	− 1.8	− 0.84
*P*	0.25	0.35	0.076	0.40

### Health regions

*Dermacentor* variabilis submission rates per 100,000 population were highest in the Central West (312) and South West (306) regions (Supplementary Fig. S1). The fastest increases in *D*. *variabilis* submissions were from the North East (RR = 1.4, 95% CI: 1.29–1.42) and Central East (RR = 1.3, 95% CI: 1.27–1.32) regions; a RR of 1.4 corresponds to an average annual increase of 40% in submission counts (Supplementary Fig. S2). There was an annual 10% decrease in submission counts from the Eastern region (RR = 0.9, 95% CI: 0.86–0.90).

### Public health units

**Figure 2:**
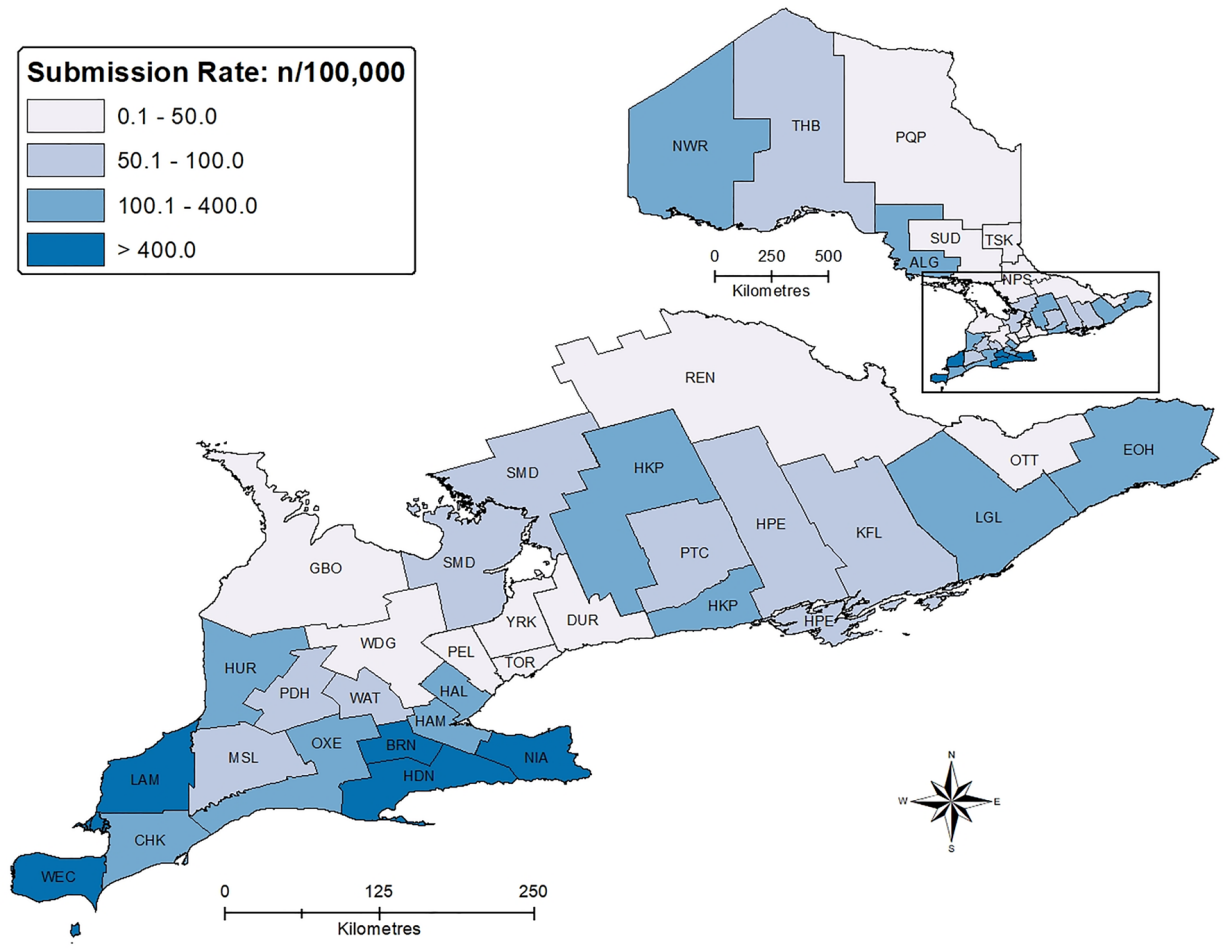
*Dermacentor* *variabilis* submission rates per 100,000 population, by public health unit: Ontario, Canada (2010–2018).

The highest *D*. variabilis submissions per 100,000 population were from LAM (902), HDN (816), NIA (682), BRN (628) and WEC (586) (Fig. 2, Supplementary Table S1). Multiple *D*. *variabilis* (≥ 2 ticks in one submission) submissions per 100,000 population were highest in the same PHUs: LAM (66), HDN (45), BRN (39), NIA (37) and WEC (24).

**Figure 3:**
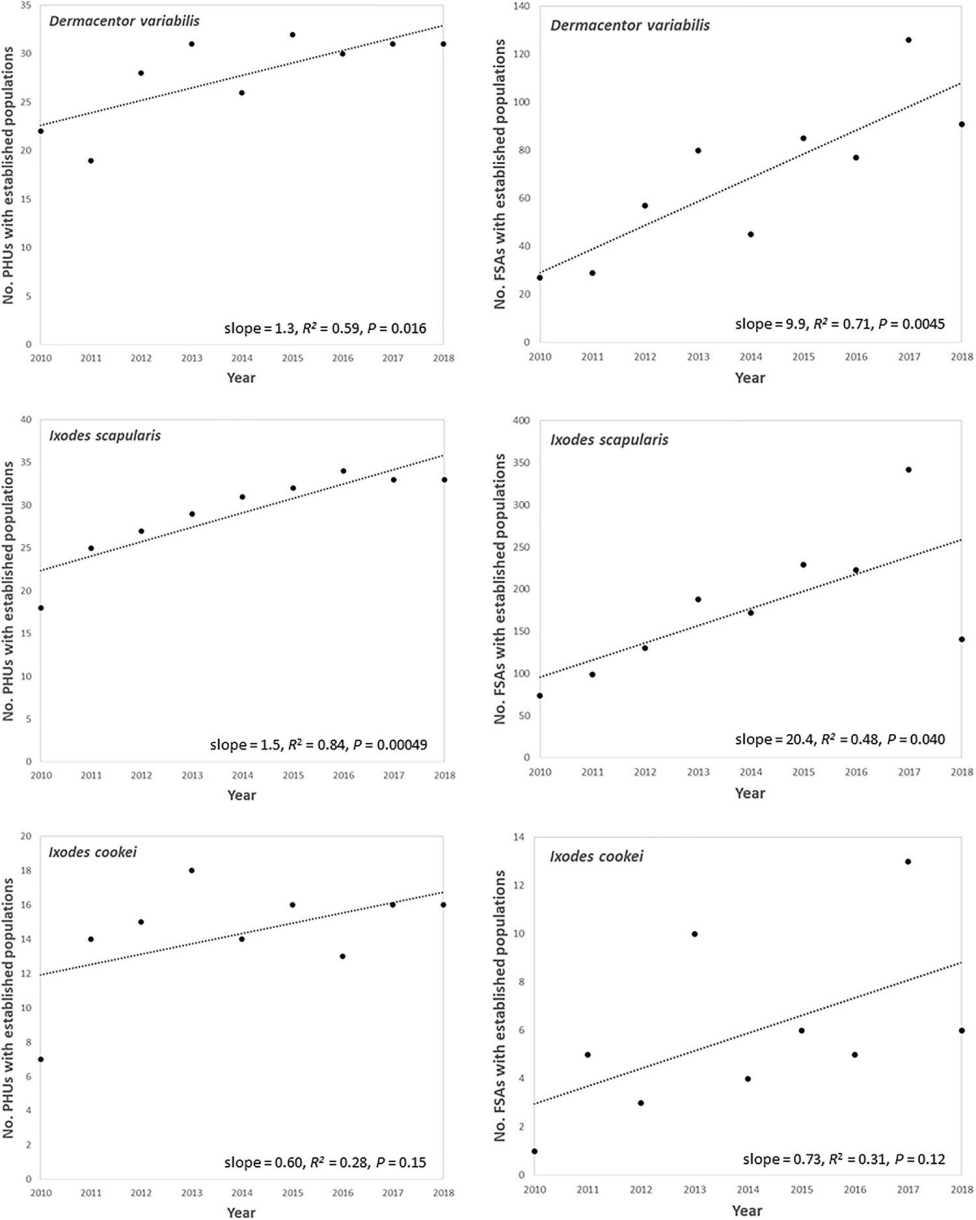
Relationship between *Dermacentor* *variabilis* submissions and time by public health unit (PHU, left panel) and Forward Sortation Area (FSA, right panel), compared with *Ixodes* *scapularis* (positive control) and *Ixodes* *cookei* (negative control): Ontario, Canada (2010–2018).

There were significant increases in the number of PHUs with an established *D*. *variabilis* population from 2010 through 2018 (*R*^2^ = 0.59, *P* = 0.016) (Fig. 3). The number of PHUs with an established *I*. *scapularis* (positive control) population increased significantly during the study (*R*^2^ = 0.84, *P* = 0.00049), while there was no change for *I*. *cookei* (negative control, *R*^2^ = 0.28, *P* = 0.15). The fastest increase in American dog tick submissions was from ALG (RR = 1.5, 95% CI: 1.37–1.56), with relatively fast increases in HAL, PTC, REN, HKP and WEC (RR = 1.3–1.4) (Supplementary Fig. S3).

### Forward sortation areas

FSAs were available for 84.5% (14,729/17,434) of submissions. FSAs with relatively higher *D*. *variabilis* counts (≥ 12 submissions per year) were primarily located in the Central West (FSAs for BRN, HAM and NIA: L0R, L0S, L2A, L3C, L3K, L3M, L8E, N0A, N0E, N1A, N3R, N3S, N3T, N3Y) and South West (FSAs for LAM and WEC: N0N, N0R, N7S, N7T, N8H, N8M, N9E, N9J, N9V, N9Y) regions (data not shown).

The number of FSAs with an established *D*. *variabilis* population increased significantly during the study (*R*^2^ = 0.71, *P* = 0.0045) (Fig. 3). The number of FSAs with an established *I*. *scapularis* (positive control) population increased significantly (*R*^2^ = 0.48, *P* = 0.040), while there was no change for *I*. *cookei* (negative control, *R*^2^ = 0.31, *P* = 0.12) (Fig. 3). There were significant increases in the number of *D*. *variabilis*-positive FSAs (at least one submission per year = occurrence) during the study (*R*^2^ = 0.73, *P* = 0.0036), higher compared to *I*. *scapularis* (positive control, *R*^2^ = 0.56, *P* = 0.20) and *I*. *cookei* (negative control, *R*^2^ = 0.46, *P* = 0.045) (Supplementary Fig. S4). There was a 10% annual increase in the number of *D*. *variabilis*-positive FSAs (RR = 1.1, 95% CI: 1.09–1.13) (Supplementary Fig. S5).

## Discussion

*Dermacentor* *variabilis* is a potential vector of several pathogens of public and/or veterinary health concern and, in Ontario, is expanding its range from key hotspots of relatively high abundance. Ontarians submitted *D*. *variabilis* from all health regions and PHUs of the province, with the highest submission rates from the Central West (BRN, HDN and NIA) and South West (LAM and WEC) regions. In addition, these hot spots experienced the highest multiple-tick submission rates, suggesting higher densities of ticks that potentially act as sources of expansion. While not a part of our analyses, submission hot spots were in the Mixedwood Plains Ecozone; specifically, in areas with higher temperatures (> 2,200 growing-degree-days above 5 °C) and lower elevation (< 225 m)^32^. The characterization of these high-submission areas is similar to studies showing that the American dog tick favours conditions associated with low elevation (e.g., higher humidity, higher temperature), favouring expansion and establishment^10,33–35^. In neighbouring Michigan, USA, American dog ticks were more abundant along large bodies of water in low elevation areas of the state’s southeast region and Northern Peninsula, contiguous with high submission areas of Ontario^36^. In southern Maine, USA, *D*. *variabilis* were more common in warmer areas at low elevation^31^. High-submission areas in Ontario have a high-percentage of poorly-drained, gleysolic soils (associated with wetlands or where wetlands once existed), consistent with reports of increased *D*. *variabilis* abundance in high-humidity habitats with damp soils^32,37,38^. There is an opportunity to model suitable habitat for *D*. *variabilis* in Ontario at a finer geographic scale (i.e., FSA), using passive and active surveillance data under current and future climate projections. 

Submission rates and counts for *D*. *variabilis* are increasing in Ontario; however, we did not confirm increasing relative abundance at the provincial level during the study. We received approximately 2,400 *D*. *variabilis* annually during this study (2013–2018), higher than previous passive surveillance work in Ontario. From 2008 through 2012, there were approximately 1,000 submissions per year in the province, and about 450 submissions per year from 1999 through 2007 (MPN unpublished data)^6^. A 1975 report noted the collection of eight *D*. *variabilis* from humans and dogs in southern Ontario from 1957 through 1975^39^. In southern Ontario from 1967 through 1977, 21 of 65 tick submissions to public health for identification were American dog ticks^19^. Lindquist et al. (2016) reported that *D*. *variabilis* is “locally abundant in southern Québec and Ontario”^2^. While passive surveillance was not able to detect increased *D*. *variabilis* relative abundance at the provincial level, abundance changes at the local level may well be determined in the future using active surveillance (e.g., tick dragging).

Undoubtedly, range expansion of *D*. *variabilis* was in progress for years to decades prior to our study, yet we demonstrate expansion in Ontario over a short period of 9 years and provide a baseline for future comparisons. The number of PHUs with established *D*. *variabilis* populations increased from 22 to 31 during the study, indicating range expansion at the regional level. We note that the distribution of ticks within an established PHU is likely heterogeneous. At the local level, the number of FSAs with established American dog tick populations increased from 27 to 91 and the number of *D*. *variabilis*-positive FSAs (occurrence) increased from 161 to 315. The difference in FSAs between occurrence (at least 1 tick) and establishment (6 or more ticks per year) may represent areas of Ontario where pioneer *D*. *variabilis* encounter unfavorable habitat and climate and fail to establish. Furthering our conclusion of range expansion, the number of PHUs and FSAs with established *D*. *variabilis* populations increased over time in a manner similar to the positive control (*I*. *scapularis*); in contrast, there was no change in the distribution of the negative control (*I*. *cookei*). Range expansion of American dog ticks in Ontario agrees with similar observations of expansion in Canada (Manitoba, Saskatchewan) and the USA (Maine, Pennsylvania)^21,31,40^. *Dermacentor* *variabilis* range expansion indicates conditions are favourable for this tick across an area larger than they currently occur in Ontario (continued expansion), potentially increasing threats to public and veterinary health. 

Relatively fast increases in *D*. *variabilis* submissions identified areas where the ticks are emerging or continuing to proliferate. The greatest increase in *D*. *variabilis* submissions over the study period were in the North East region, which was unexpected given the region consisted mostly of PHUs without established tick populations. Submissions increased annually by almost 50% in ALG, the PHU driving the rapid increase in the North East. The recent increase in *D*. *variabilis* in ALG (centered in and around Sault Ste. Marie) is likely the result of expansion of *D*. *variabilis* populations from the Upper Peninsula of Michigan, or possibly successful introduction and establishment of adventitious ticks from southern Ontario. We presume *D*. *variabilis* dispersal in agricultural southern Ontario occurs via host movement among patches of suitable habitat or stepping stones (e.g., isolated woodlots within cultivated land)^41^. The variability in the rate of increase of submissions by PHU may reflect variability in the size and proximity of these stepping stones (slower where stepping stones are widespread and small), in conjunction with variability in suitable climate and ecological requirements. Geographic expansion of *D*. *variabilis* in Ontario does not represent diffusion from a single historical population, rather a mosaic of hot spots expanding at variable rates toward areas with favourable habitat and climate. 

While not specifically examined in this study, the range expansion of the American dog tick is likely due to a combination of climate change (e.g., higher annual temperatures and milder winters), changes in land use patterns (e.g., succession of abandoned agricultural land and forest fragmentation), changes in host distribution/abundance (e.g., white-tailed deer, *Odocoileus* *virginianus*) and increased human travel (including dog travel). Increasing temperature is an important driver for the range expansion of *D*. *variabilis* and other ticks in North America, such as the blacklegged tick and the lone star tick^30,42^. Worldwide, other tick species are experiencing range expansions, such as *I.* *ricinus* in Northern Europe and *Dermacentor* *reticulatus* in Central Europe^43,44^. *Dermacentor* *variabilis* is hypothetically expanding along waterways found in high-submission areas of Ontario, similar to American dog tick expansion in Saskatchewan, Canada, along the South Saskatchewan River and Diefenbaker Lake^21^. For example, hosts travelling along waterways of the Grand River Watershed in Ontario’s Central West region potentially aid the gradual dispersal of adult *D*. *variabilis*, especially host animals with relatively larger home ranges such as coyotes, red foxes (*Vulpes* *vulpes*) and white-tailed deer. In contrast to other ixodid ticks, American dog ticks rarely feed on birds, limiting long-distance and rapid dispersal^2^. Rapid and long-distance dispersal of *D*. *variabilis* is usually via anthropogenic means, whereby adventitious ticks travel on humans, companion animals or livestock. There is an opportunity to test hypotheses underpinning *D*. *variabilis* expansion in Ontario using active and passive surveillance, which can aid in predicting the location of potential emerging populations. 

Adult *D*. *variabilis* activity in Ontario peaked from late May through early June, similar to elsewhere in the northern portion of the tick’s range; e.g., May (Pennsylvania), late May through early June (Maine, Nova Scotia) and June (Quebec)^31,38,45,46^. The life cycle of the American dog tick normally takes two years in northern latitudes, longer depending on local host availability and climate^2,47^. In warmer climates, *D*. *variabilis* has a one-year life cycle with longer activity periods and multiple adult cohorts with two (Kentucky, USA) or three (Georgia, USA) activity peaks during the spring and summer^48,49^. It will be interesting to see if there will be earlier-season shifts in *D*. *variabilis* activity in North America due to warming temperatures. 

The public and veterinary health importance of American dog ticks in Ontario deserves attention, especially since we have identified population hot spots and expansion. Rocky Mountain spotted fever and tularemia are the primary tick-borne disease threats posed by American dog ticks in Ontario. Rocky Mountain spotted fever is not reportable to public health officials in Ontario; however, researchers in 1978 described a locally acquired case near Ottawa, the only documented case acquired in Ontario^50^. In a 2006 report, a dog from Ottawa, with no history of travel, was seropositive for *R*. *rickettsii*^51^. Tularemia is endemic throughout Ontario; however, human infection is usually the result of skinning infectious animals (e.g., common muskrats, *Ondatra* *zibethicus*; eastern cottontails, *Sylvilagus* *floridanus*), rather than tick-borne transmission^52^. Tularemia is reportable to public health in Ontario, however, it is rare, with only six cases reported in Ontario from 2005 through 2019^53^. Researchers detected *F*. *tularensis* and *R*. *rickettsii* in American dog ticks in Ontario from the 1960s into the 1980s^17,54^. In recently published studies (2016–2018), screening of *D*. *variabilis* from Ontario revealed the presence of *Rickettsia* *montanensis* [spotted fever group rickettsiae (SFGR) with unknown pathogenicity] and *Rickettsia* *peacockii* (non-pathogenic, endosymbiotic SFGR)^55,56^. We infer that the risk of *D*. *variabilis*-borne disease in Ontario is low; however, the epidemiological significance of the American dog tick in the province is underexplored. 

There are several caveats to our study, which are associated with any surveillance program based on the passive submission of specimens. The location of tick acquisition is not always the same as the submitter’s place of residence; although we excluded ticks where the submitter indicated that they were acquired outside of Ontario. We assumed that most tick exposures occurred near the submitter’s home; therefore, we did not take into account possible human travel within Ontario. Increased awareness of the surveillance program potentially contributed to American dog ticks being submitted from a wider geographic area; however, the extent of this awareness and its impact on submissions is unknown. We also assumed tick submission behaviour did not vary among PHUs; though, the public in areas accustomed to this tick may not have sought identification. In addition, PHUs potentially did not submit all *D*. *variabilis* for identification, as they are relatively easy to identify. Our surveillance system did not include submissions from non-human hosts, meaning important hosts were missed (e.g., dogs); future work including collections from companion animals may elucidate further information on the distribution of American dog ticks in Ontario. We acknowledge that the 6-tick criterion for deeming a PHU or FSA as having an established population is a proxy until Ontario- and species-specific benchmarks are developed; this criterion likely overestimates the number of “true established populations” in the absence of confirmatory tick dragging. Further work is needed to determine if the 6-tick criterion is applicable at multiple scales. We should note that additional research is needed to quantify the extent that Ontario population growth (approximately 12.9 million in 2011 and 13.4 million in 2016) and loss of farm land (approximately 64.8 million hectares in 2011 and 64.2 million hectares in 2016) contributes to the spread of the American dog ticks in Ontario, especially at the FSA level^57,58^. The impact of discontinued passive surveillance meant it was not possible to assess spatiotemporal changes in *D*. *variabilis* populations in the Eastern region and we possibly missed areas of high submission rates or areas with rapidly expanding populations. 

## Conclusions

We have provided a detailed account of *D*. *variabilis* distribution and range expansion at multiple spatial scales in Ontario and we suggest continued research into American dog tick populations and their associated pathogens. In addition, further ecological work will add to our understanding of the factors contributing to *D*. *variabilis* emergence and expansion. 

## Supplementary Information

Supplementary Information.

## Data Availability

Information about PHO’s data access request process is available on-line at: https://www.publichealthontario.ca/en/Data-and-Analysis/Using-Data/Data-Requests.
