# Supplementary material for: American dog ticks along their expanding range edge in Ontario, Canada
Source: Sci Rep. 2022 Jun 30;12:11063. doi: 10.1038/s41598-022-15009-9 (PMC9247098; doi:10.1038/s41598-022-15009-9)

Supplementary Material

Title

American dog ticks along their expanding range edge in Ontario, Canada

Running head

Dermacentor variabilis spread in Ontario

Authors

Mark P. Nelder[1*], Curtis B. Russell[1], Steven Johnson[2], Ye Li[2,3], Kirby Cronin[4,5], Tania Cawston[4] & Samir N. Patel[4,6]

Affiliations

[1]Enteric, Zoonotic and Vector-Borne Diseases; Health Protection, Operations and Response; Public Health Ontario; Toronto, Ontario, Canada

[2]Informatics, Knowledge Services, Public Health Ontario, Toronto, ON, Canada

[3]Dalla Lana School of Public Health, University of Toronto, Toronto, ON, Canada

[4]Public Health Ontario Laboratory, Public Health Ontario, Toronto, ON, Canada

[5]National Microbiology Laboratory, Public Health Agency of Canada, Winnipeg, Manitoba, Canada

[6]Department of Laboratory Medicine and Pathobiology, University of Toronto, Toronto, ON, Canada

[*]e-mail: mark.nelder@oahpp.ca

Supplementary Table S1. Summary of *Dermacentor variabilis* submissions by public health unit: Ontario, Canada (2010–2018). EOH, HDN, KFL (2014) and LGL (2018) ceased passive tick submissions to their offices during the study.

Public health unit (acronym)	Public health unit (full name)	Total *D. variabilis* submissions	*D. variabilis* submission rate per 100,000 population	*D. variabilis* multiple tick submission rate per 100,000 population
**ALG**	Algoma District	206	179	15
**BRN**	Brant County	924	628	39
**CHK**	Chatham-Kent	408	388	8
**DUR**	Durham Regional	183	27	2
**EOH**	Eastern Ontario	358	174	8
**GBO**	Grey Bruce	82	50	2
**HAL**	Halton Regional	1,095	192	9
**HAM**	City of Hamilton	1,506	268	9
**HDN**	Haldimand-Norfolk	1,095	984	45
**HKP**	Haliburton-Kawartha-Pine Ridge District	508	280	15
**HPE**	Hastings and Prince Edward Counties	97	59	1
**HUR**	Huron County	73	123	2
**KFL**	Kingston-Frontenac and Lennox & Addington	135	66	3
**LAM**	Lambton	1,170	902	66
**LGL**	Leeds-Grenville and Lanark District	548	324	14
**MSL**	Middlesex-London	308	65	3
**NIA**	Niagara Regional	3,093	682	37
**NPS**	North Bay Parry Sound District	46	36	2
**NWR**	Northwestern	144	177	7
**OTT**	City of Ottawa	146	15	<1
**OXE**	Oxford Elgin-St. Thomas	446	219	5
**PDH**	Perth District	78	99	4
**PEL**	Peel Regional	287	20	1
**PQP**	Porcupine	8	9	0
**PTC**	Peterborough County-City	119	84	4
**REN**	Renfrew County and District	19	18	0
**SMD**	Simcoe Muskoka District	336	60	2
**SUD**	Sudbury and District	52	26	0
**THB**	Thunder Bay District	123	80	2
**TOR**	City of Toronto	498	17	1
**TSK**	Timiskaming	7	21	3
**WAT**	Waterloo	543	97	3
**WDG**	Wellington-Dufferin-Guelph	142	48	2
**WEC**	Windsor-Essex County	2,418	586	24
**YRK**	York Regional	233	20	1
**Provincial**		17,434	124	5.7

Supplemental Model Outputs.

Modelling submissions on Health Region level:

Call: glm(formula = N ~ sin1 + cos1 + sin2 + cos2 + Year * HU.Region, family = "poisson", data = HRD, offset = logPop)

Deviance Residuals:

Min	1Q	Median	3Q	Max
-12.2049	-1.0715	-0.1862	-0.0253	13.2058

**Coefficients:**	Estimate	Std. Error	z value	Pr(>|z|)
(Intercept)	533.682	21.16688	-25.213	<2e-16***
sin1	-0.36666	0.05145	-7.126	1.03e-12***
cos1	-5.10352	0.15438	-33.058	<2e-16***
sin2	-0.56591	0.03487	-16.229	<2e-16***
cos2	-0.24529	0.05671	-4.326	1.52e-05***
Year	0.25709	0.01050	24.482	<2e-16***
HU.RegionCentral West	199.07049	22.87030	8.704	<2e-16***
HU.RegionEastern	770.08655	30.12709	25.561	<2e-16***
HU.RegionNorth East	-89.17386	53.17663	-1.677	0.0936.
HU.RegionNorth West	287.34254	52.41635	5.482	4.21e-08***
HU.RegionSouth West	206.76793	23.74223	8.709	<2e-16***
HU.RegionToronto	193.44273	41.28327	4.686	2.79e-06***
Year:HU.RegionCentral West	-0.09776	0.01135	-8.615	<2e-16***
Year:HU.RegionEastern	-0.38200	0.01496	-25.542	<2e-16***
Year:HU.RegionNorth East	0.04442	0.02638	1.684	0.0922.
Year:HU.RegionNorth West	-0.14210	0.02601	-5.462	4.70e-08***
Year:HU.RegionSouth West	-0.10156	0.01178	-8.621	<2e-16***
Year:HU.RegionToronto	-0.09639	0.02049	-4.705	2.53e-06***

Signif. codes: 0 ‘***’ 0.001 ‘**’ 0.01 ‘*’ 0.05 ‘.’ 0.1 ‘ ’ 1

(Dispersion parameter for poisson family taken to be 1)

Null deviance: 69555.0 on 755 degrees of freedom

Residual deviance: 4593.4 on 738 degrees of freedom

AIC: 6184.8

Number of Fisher Scoring iterations: 7

Modelling submissions on Health Unit level:

Call: glm(formula = N ~ sin1 + cos1 + sin2 + cos2 + Year * Health.Unit, family = "poisson", data = PHUD, offset = logPop)

Deviance Residuals:

Min	1Q	Median	3Q	Max
-7.3431	-0.6283	-0.1157	-0.0220	17.0221

**Coefficients:**	Estimate	Std. Error	z value	Pr(>|z|)
(Intercept)	-779.826	64.77640	-12.039	<2e-16***
sin1	-0.36136	0.05179	-6.977	3.02e-12***
cos1	-5.09142	0.15506	-32.835	<2e-16***
sin2	-0.56627	0.03510	-16.132	<2e-16***
cos2	-0.24075	0.05700	-4.224	2.40e-05***
Year	0.37997	0.03213	11.826	<2e-16***
Health.UnitBRN	309.87539	70.20630	4.414	1.02e-05***

93	Health.UnitCHK	332.89325	76.91569	4.328	1.50e-05***
94	Health.UnitDUR	280.92354	89.76415	3.130	0.001751**
95	Health.UnitEOH	947.12564	76.33224	12.408	<2e-16***
96	Health.UnitGBO	481.89134	109.35473	4.407	1.05e-05***
97	Health.UnitHAL	101.71958	70.72490	1.438	0.150365
98	Health.UnitHAM	391.50164	68.12994	5.746	9.12e-09***
99	Health.UnitHDN	405.66657	69.22352	5.860	4.62e-09***
100	Health.UnitHKP	150.31081	75.86819	1.981	0.047568*
101	Health.UnitHPE	480.82288	104.47286	4.602	4.18e-06***
102	Health.UnitHUR	446.90231	115.16578	3.881	0.000104***
103	Health.UnitKFL	1032.28313	93.19357	11.077	<2e-16***
104	Health.UnitLAM	787.77511	68.19054	11.553	<2e-16***
105	Health.UnitLGL	1315.57635	74.45329	17.670	<2e-16***
106	Health.UnitMSL	602.28562	77.74733	7.747	9.43e-15***
107	Health.UnitNIA	600.17397	66.16769	9.070	<2e-16***
108	Health.UnitNPS	405.41791	135.06794	3.002	0.002686**
109	Health.UnitNWR	499.49245	91.93787	5.433	5.54e-08***
110	Health.UnitOTT	569.37592	90.83031	6.269	3.64e-10***
111	Health.UnitOXE	430.20420	70.29029	6.120	9.33e-10***
112	Health.UnitPDH	565.43970	105.66024	5.351	8.72e-08***
113	Health.UnitPQP	522.74340	290.89946	1.797	0.072337.
114	Health.UnitPTC	96.94472	106.17692	0.913	0.361217
115	Health.UnitREN	95.58888	228.65887	0.418	0.675917
116	Health.UnitSMD	318.15317	79.37154	4.008	6.11e-05***
117	Health.UnitSUD	357.20880	133.99947	2.666	0.007682**
118	Health.UnitTHB	572.71404	95.95379	5.969	2.39e-09***
119	Health.UnitTOR	439.59458	73.83937	5.953	2.63e-09***
120	Health.UnitTSK	686.91112	268.50518	2.558	0.010519*
121	Health.UnitWAT	488.42778	73.25804	6.667	2.61e-11***
122	Health.UnitWDG	356.75943	95.52626	3.735	0.000188***
123	Health.UnitWEC	240.47031	67.14547	3.581	0.000342***
124	Health.UnitYRK	285.21307	85.67628	3.329	0.000872***
125	Year:Health.UnitBRN	-0.15311	0.03482	-4.397	1.10e-05***
126	Year:Health.UnitCHK	-0.16482	0.03815	-4.320	1.56e-05***
127	Year:Health.UnitDUR	-0.14031	0.04453	-3.151	0.001627**
128	Year:Health.UnitEOH	-0.47011	0.03787	-12.412	<2e-16***
129	Year:Health.UnitGBO	-0.23977	0.05426	-4.419	9.92e-06***
130	Year:Health.UnitHAL	-0.05045	0.03508	-1.438	0.150371
131	Year:Health.UnitHAM	-0.19406	0.03379	-5.743	9.33e-09***
132	Year:Health.UnitHDN	-0.20046	0.03434	-5.838	5.28e-09***
133	Year:Health.UnitHKP	-0.07436	0.03763	-1.976	0.048144*
134	Year:Health.UnitHPE	-0.23917	0.05184	-4.614	3.95e-06***
135	Year:Health.UnitHUR	-0.22198	0.05714	-3.885	0.000102***
136	Year:Health.UnitKFL	-0.51288	0.04626	-11.087	<2e-16***
137	Year:Health.UnitLAM	-0.39013	0.03383	-11.534	<2e-16***
138	Year:Health.UnitLGL	-0.65285	0.03695	-17.671	<2e-16***
139	Year:Health.UnitMSL	-0.29936	0.03857	-7.761	8.41e-15***
140	Year:Health.UnitNIA	-0.29715	0.03282	-9.054	<2e-16***

Year:Health.UnitNPS	-0.20197	0.06702	-3.014	0.002582**
Year:Health.UnitNWR	-0.24786	0.04562	-5.434	5.52e-08***
Year:Health.UnitOTT	-0.28376	0.04507	-6.296	3.05e-10***
Year:Health.UnitOXE	-0.21305	0.03487	-6.110	9.94e-10***
Year:Health.UnitPDH	-0.28084	0.05243	-5.356	8.49e-08***
Year:Health.UnitPQP	-0.26090	0.14438	-1.807	0.070756.
Year:Health.UnitPTC	-0.04849	0.05267	-0.921	0.357190
Year:Health.UnitREN	-0.04863	0.11342	-0.429	0.668122
Year:Health.UnitSMD	-0.15841	0.03937	-4.023	5.74e-05***
Year:Health.UnitSUD	-0.17824	0.06648	-2.681	0.007342**
Year:Health.UnitTHB	-0.28463	0.04761	-5.978	2.26e-09***
Year:Health.UnitTOR	-0.21927	0.03663	-5.986	2.15e-09***
Year:Health.UnitTSK	-0.34187	0.13330	-2.565	0.010325*
Year:Health.UnitWAT	-0.24267	0.03634	-6.678	2.43e-11***
Year:Health.UnitWDG	-0.17768	0.04739	-3.749	0.000177***
Year:Health.UnitWEC	-0.11874	0.03330	-3.565	0.000363***
Year:Health.UnitYRK	-0.14260	0.04250	-3.355	0.000793***

Signif. codes: 0 '***' 0.001 '**' 0.01 '*' 0.05 '.' 0.1 ' ' 1

(Dispersion parameter for poisson family taken to be 1)

Null deviance: 84505.5 on 3671 degrees of freedom

Residual deviance: 7462.7 on 3600 degrees of freedom

AIC: 11941

Number of Fisher Scoring iterations: 7

<u>Modelling submissions on Forward Sortation Area (FSA) level:</u>

Call: glm(formula = NN ~ sin1 + cos1 + sin2 + cos2 + Year, family = "poisson", data = FSAD)

Deviance Residuals:

Min	1Q	Median	3Q	Max
-7.5291	-1.6417	-0.6833	0.7622	7.9911

Coefficients: Estimate Std. Error z value Pr(>|z|)

(Intercept)	2.927e+02	9.260e+01	3.161	0.00157**
sin1	-2.866e+02	6.677e+01	-4.292	1.77e-05***
cos1	-6.314e+02	1.042e+02	-6.057	1.39e-09***
sin2	1.567e+02	2.874e+01	5.451	5.01e-08***
cos2	1.315e+02	1.378e+01	9.544	<2e-16***
Year	9.989e-02	5.512e-03	18.120	<2e-16***

Signif. codes: 0 '***' 0.001 '**' 0.01 '*' 0.05 '.' 0.1 ' ' 1

(Dispersion parameter for poisson family taken to be 1)

Null deviance: 10333.36 on 107 degrees of freedom

Residual deviance: 650.33 on 102 degrees of freedom

AIC: 1034.4

Number of Fisher Scoring iterations: 6

Supplementary Table S2. Summary of *Dermacentor variabilis*, *Ixodes scapularis* and *Ixodes cookei* submissions: Ontario, Canada (2010–2018). **All:** data includes all public health units (PHUs). **Subset:** data excludes PHUs that ceased tick submissions during the study; i.e., EOH, HDN, KFL and LGL.

Year	Tick submissions per year							
	Dermacentor variabilis		*Ixodes scapularis*		*Ixodes cookei*		All tick species	
	**All**	**Subset**	**All**	**Subset**	**All**	**Subset**	**All**	**Subset**
**2010**	773	588	996	310	147	103	2,042	1,102
**2011**	910	784	2,298	641	227	152	3,568	1,683
**2012**	1,519	1,130	2,521	718	198	140	4,444	2,144
**2013**	2,089	1,704	3,021	969	289	208	6,021	3,224
**2014**	1,129	964	2,117	1,229	241	209	4,091	2,829
**2015**	2,446	2,306	1,890	1,579	270	262	5,142	4,627
**2016**	1,901	1,728	2,041	1,752	199	197	4,629	4,177
**2017**	4,095	3,734	4,559	4,020	398	395	9,671	8,851
**2018**	2,572	2,360	3,469	3,206	355	352	6,833	6,393
**Total**	17,434	15,298	22,912	14,424	2,324	2,018	46,441	35,030
**Median (IQR)**	1,901 (1,020–2,509)	1,704 (874–2,333)	2,298 (1,966–3,245)	1,299 (680–2,479)	241 (199–322)	208 (146–307)	4,629 (3,830–6,427)	3,224 (1,914–5,510)

Supplemental Figure S1. *Dermacentor variabilis* submission rates per 100,000 population, by health region: Ontario, Canada (2010–2018). Central East (39.6), Central West (311.6), Eastern (71.5), North East (56.9), North West (113.5), South West (305.8) and Toronto (17.3)

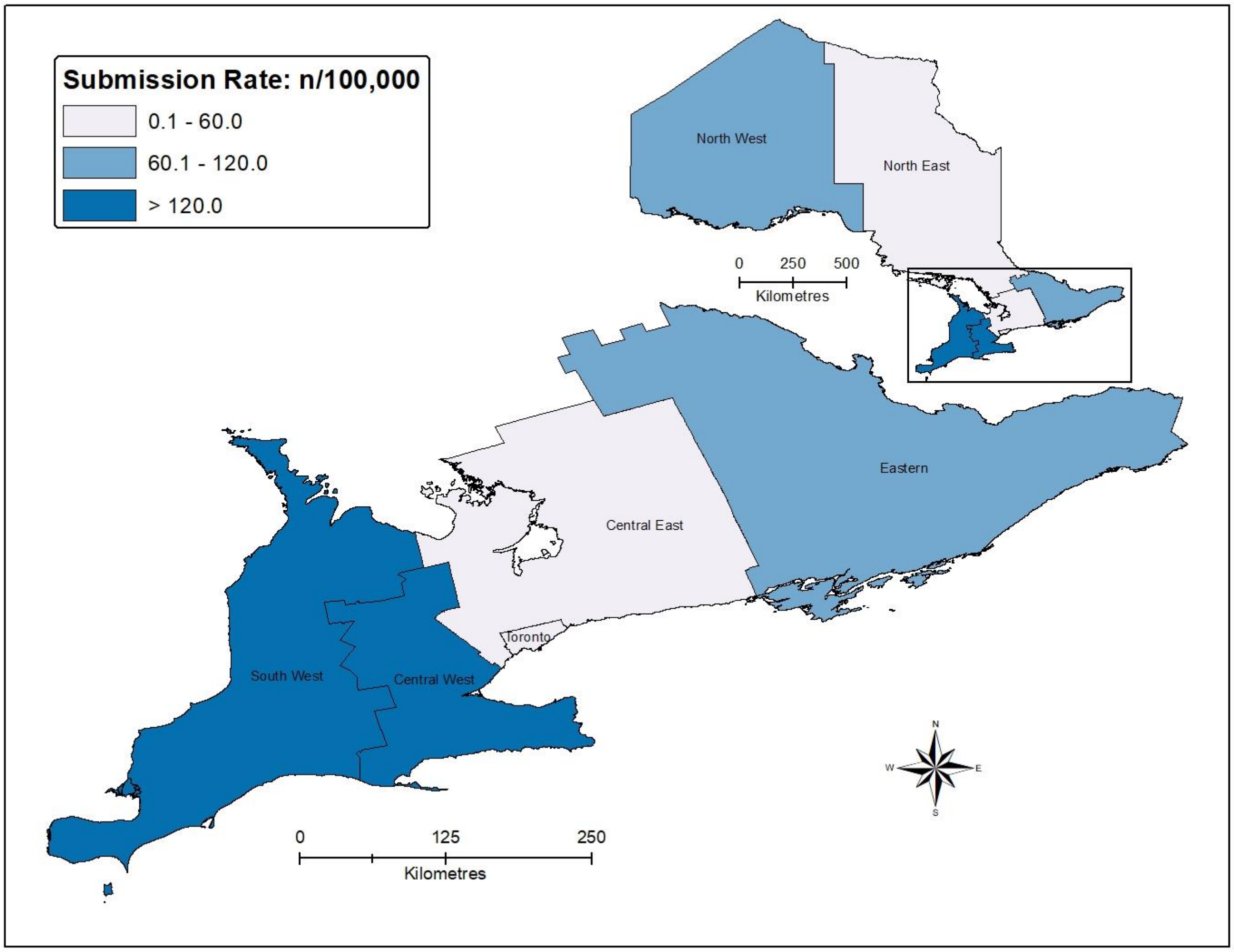

Supplementary Figure S2. Rate of increase (relative rate, 95% confidence interval) in *Dermacentor variabilis* submission counts by health region: Ontario, Canada (2010–2018).

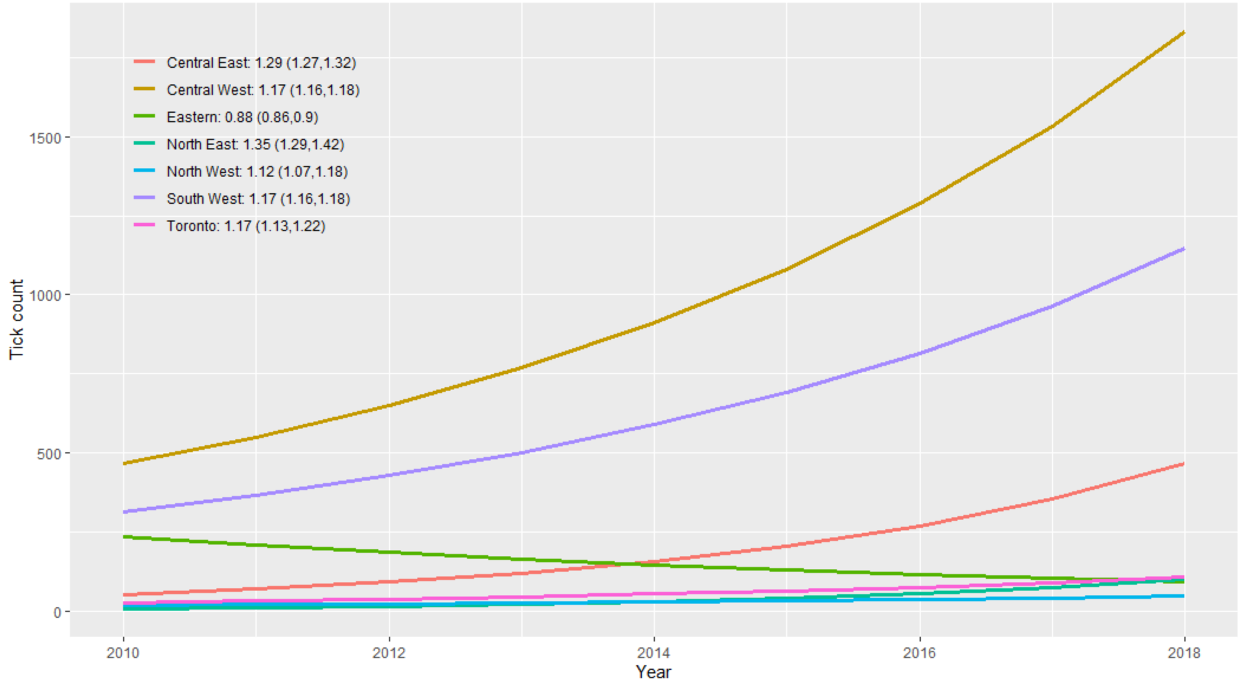

Supplementary Figure S3. Rate of increase (relative rate, 95% confidence interval) in *Dermacentor variabilis* submission counts for public health units (PHUs): Ontario, Canada (2010–2018). Figure includes top-six PHUs with fastest rates of increase, all other PHUs are indicated by grey lines.

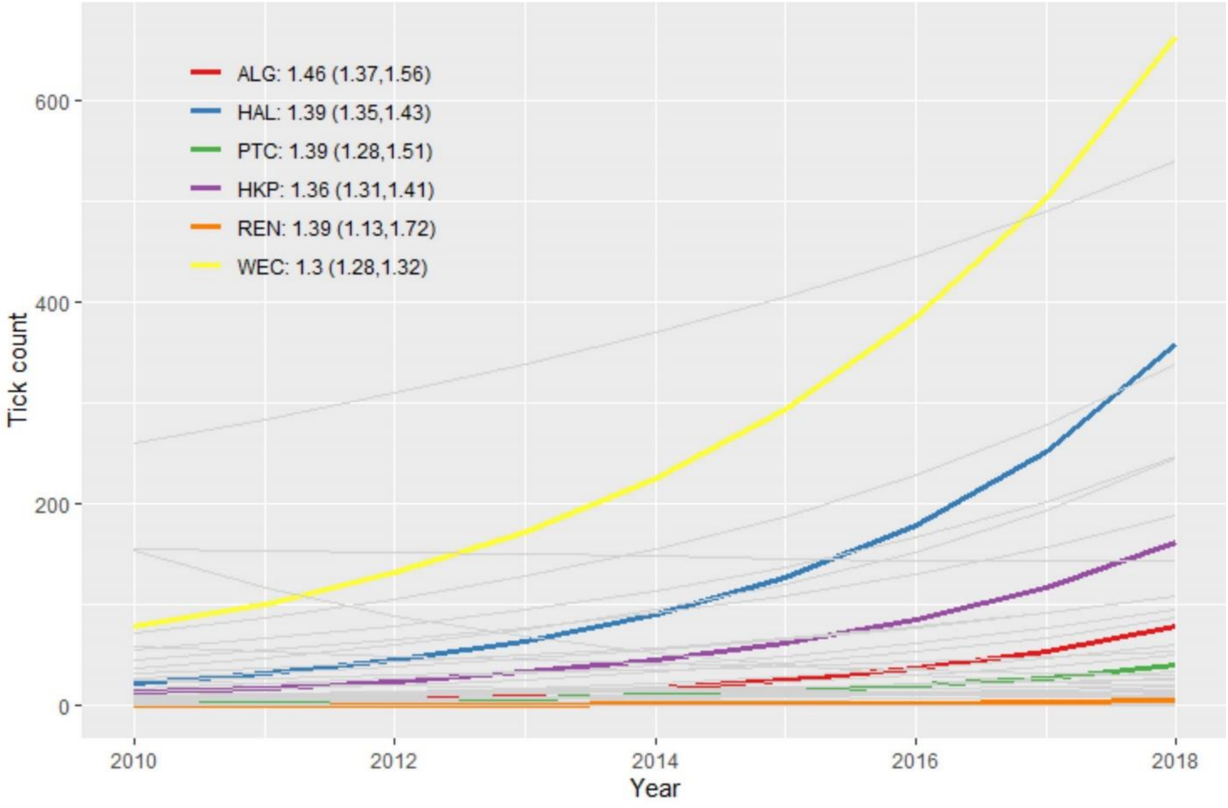

Supplementary Figure S4. Forward Sortation Areas (FSAs) positive for *Dermacentor variabilis*, *Ixodes scapularis* and *Ixodes cookei* over time: Ontario, Canada (2010–18)

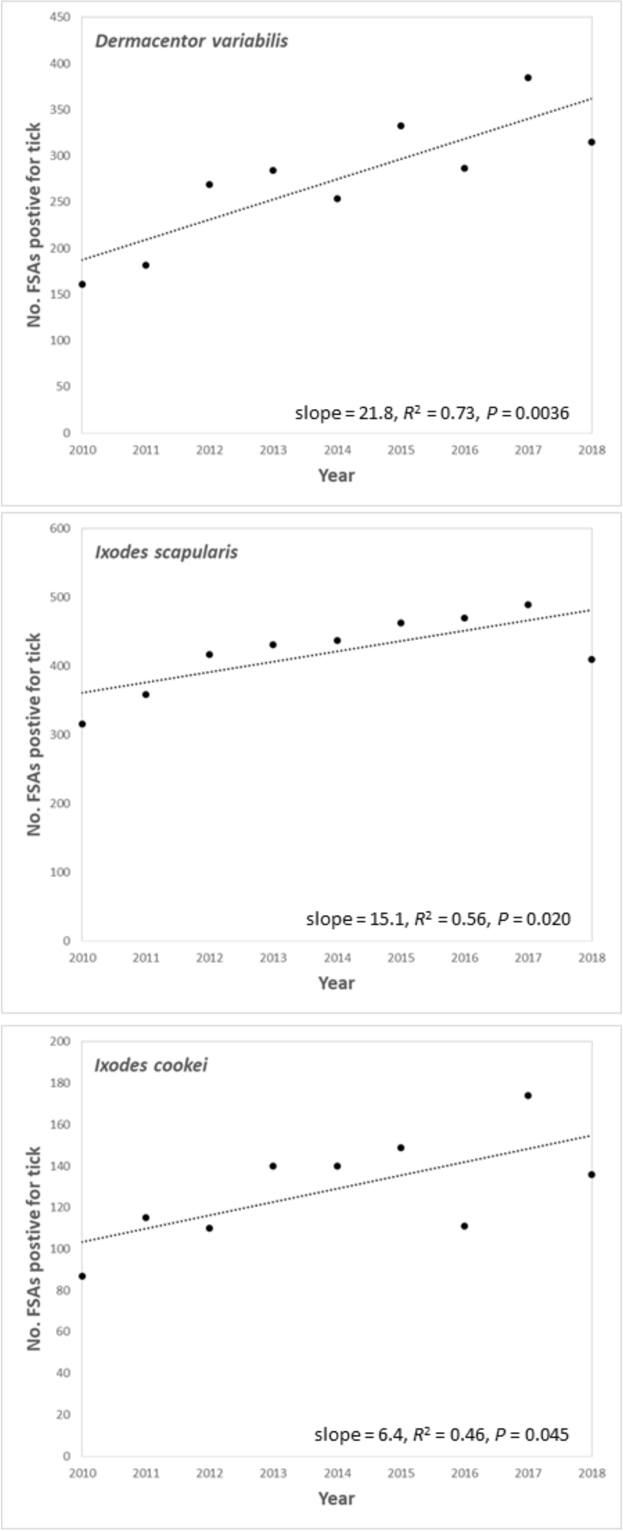

Supplementary Figure S5. Rate of increase (relative rate, 95% confidence interval) in number of *Dermacentor variabilis*-positive FSAs: Ontario, Canada (2010–2018).

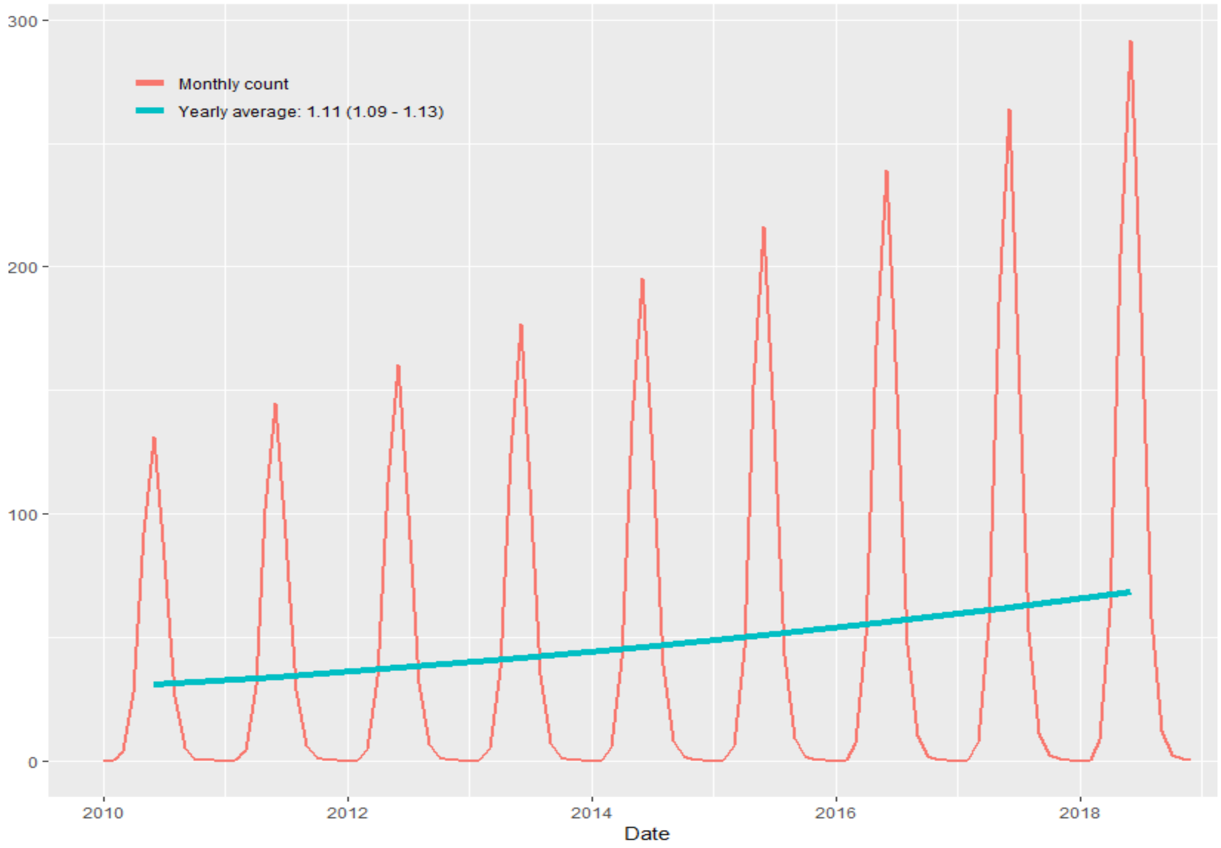

Supplement: Supplementary file 1 — Supplementary Information. [file 41598_2022_15009_MOESM1_ESM.pdf]
